# Surface Electrical Impedance Myography in Assessment of Morphofunctional Changes in Biological Tissues and Biofeedback Interfaces

**DOI:** 10.3390/s26154926

**Published:** 2026-08-04

**Authors:** Vladislava Kapravchuk, Andrey Briko, Sergey Shchukin

**Affiliations:** Department of Medical and Technical Information Technology, Bauman Moscow State Technical University, 105005 Moscow, Russia; briko@bmstu.ru (A.B.); schookin@bmstu.ru (S.S.)

**Keywords:** electrical impedance myography, muscles, subcutaneous fat, bioimpedance, biofeedback

## Abstract

Electrical impedance myography (EIM) is a noninvasive bioimpedance technique used to assess the structural and compositional properties of muscle tissue. It involves passing a low-intensity probing current between current electrodes and recording the resulting potential difference across measuring electrodes positioned over the muscle of interest. The complexity of signal interpretation lies in the mechanisms of its generation, which are based on dynamic morphofunctional changes in muscle tissue and the skin-fat layer during contraction, which remain incompletely understood. The aim of this review is to summarize current knowledge on the application of EIM in assessing morphofunctional changes in biological tissues for diagnostic, rehabilitation, and biofeedback purposes, taking into account the EIM signal generation mechanisms. Building upon current knowledge, the paper outlines promising avenues for future research in this area. This review includes research papers, review articles published in English from 2016 to 2025, in which EIM was used for diagnostic, rehabilitation, or biofeedback purposes. The selection criteria were experimental or clinical design involving humans or animals, the presence of quantitative data linking impedance parameters to physiological tissue properties, and the use of standard measurement techniques. The review included 51 papers selected from a search of the Scopus database covering 2016–2025 and conducted in accordance with the PRISMA-ScR criteria; a total of 458 records were identified, of which 51 studies were included in the final analysis after screening and full-text assessment. A small number of publications from 2026 are cited as supplementary background and were not part of the formal screening count. The conducted scoping review showed that EIM is a promising and informative tool for non-invasive assessment of the morphofunctional conditions of skeletal muscles, demonstrates a correlation with the muscle contraction force, and can be used as an independent or additional method for diagnostic and rehabilitation purposes. Despite the promise of the method, further detailed research signal generation mechanisms is needed. This includes the assessment and isolation of the contribution of various biological tissues to the recorded EIM signal, taking into account the determination of the electrode system location over the target muscle.

## 1. Introduction

The growing popularity of bioimpedance methods for monitoring changes in muscle structure and function, particularly in the upper limbs, is the result of an expanding range of practical applications and a deeper scientific understanding of the physiological processes underlying these methods. An analysis of scientific publications over the past decade (2016–2025) shows that interest in electrical impedance myography (hereinafter EIM) is becoming a tool for assessing morphofunctional changes in biological tissues in clinical practice [1], rehabilitation [2], and the development of biofeedback systems [3,4]. Despite the significant amount of accumulated data in these areas of EIM application, significant gaps remain regarding the standardization of measurement protocols and the unification of signal interpretation under individual anatomy. The aim of this review is not only to systematize current understanding of the bioimpedance signal formation mechanisms, but also to identify key methodological gaps that currently limit the implementation of the method in clinical practice. An analysis of research from the last decade makes it possible to summarize and identify unresolved issues, including those related to identifying the contribution of various biological tissues to the recorded signal. This review of the subject area will provide a foundation for future research, thereby accelerating the development of reliable EIM-based diagnostic criteria and control algorithms for robotic devices, minimizing the risk of data misinterpretation.

The operating principle of EIM is based on the passage of a high-frequency, low-amplitude (≤10 mA) electric current through the target tissues (in particular, the skin-fat and muscle layers). In turn, changes in the composition and architecture of tissues [5], caused by disease or muscle contraction, affect the values of electrical impedance in the frequency range of the probing current [6]. The body’s tissues actually become components of an electrical circuit, and the change in current amplitude is associated mainly with the resistive behavior of the tissues, i.e., with a change in structure. The time delay of the current is associated with capacitive/reactive components, mainly with myofibril membranes, which act as circuit capacitors [7]. Thus, measuring electrical properties (e.g., the real and imaginary components of impedance, as well as the impedance modulus and phase angle) relative to the applied current over a wide frequency range can provide information about the structure and integrity of biological tissues.

The recorded impedance values are influenced not only by the internal electrical properties of tissues, such as specific electrical resistance and relative permittivity, but also by factors such as the characteristics and location of electrodes [8], electrode material [9], interelectrode distance [10], the degree of muscle contraction/fatigue [11], as well as the volume and geometric parameters of tissues located directly under the electrode system [12]. For this reason, studies of the bioimpedance signal generation mechanisms using finite element modeling to optimize the electrode system geometry and interpret measurements of recorded signals are becoming increasingly relevant [13,14]. Computational models constructed on the basis of magnetic resonance imaging (MRI)/ultrasound/3D scanning data [15,16,17] or when modeling a limb with cylindrical inclusions [18] corresponding to biological tissues on average in thickness and electrical properties, make it possible to predict how the bioimpedance signal will change in response to physiological processes. In a small exploratory forearm study, Kapravchuk et al. [19] recorded EIM alongside ultrasound while controlling electrode-system force and stepwise displacement; pressure- and muscle-action-dependent impedance changes were accompanied by changes in the thickness of the skin-subcutaneous-fat and muscle layers. Thus, tissue architecture is demonstrably associated with EIM, but direct dynamic quantification of muscle displacement beneath a fixed electrode array in adequately powered active-contraction cohorts remains limited; tissue deformation and electrode-skin loading should be treated as potential contributors rather than as direct EIM outputs [19,20,21]. This is critically important to consider when designing proportional biofeedback systems. It is also important to note that skeletal muscle tissue has a high degree of anisotropy due to its structural features [1], which must also be taken into account in finite element modeling of current propagation in biological tissues.

Electrical impedance methods allow for non-invasive assessment of muscle tissue’s internal conditions by linking quantitative changes in bioelectric signals with morphofunctional processes. These processes can be classified based on their duration and physiological nature and include fast processes, such as changes in contraction force [11] (and in the case of longer contractions, fatigue [22]), and slow structural changes, including hypertrophy [23], atrophy [24], adipose infiltration and recovery after injury [25], adaptation to the training process [3], etc. Functional changes occurring in muscles during contraction are the basis for using electrical impedance methods in control interfaces [19]. While bioimpedance methods were initially considered primarily for diagnostic purposes, they are now being successfully integrated into a wide variety of fields. In particular, studies aimed at assessing muscle strength using EIM are gaining popularity [4]. Thus, in [26], the possibility of using multi-frequency EIM to predict grip strength in adults, including a significant proportion of elderly people, was examined. EIM data were obtained from the flexor and extensor muscle groups of the forearm. Since grip strength is a key indicator of a number of neuromuscular diseases, including sarcopenia, characterized by progressive and pathological loss of muscle mass and dysfunction of skeletal muscles, the presence of a simple diagnostic method reflecting not only the external function of muscle strength (for example, as in the dynamometry method [27]), will make it possible to assess the functional condition of elderly people and monitor the dynamics of therapy taking into account the internal properties of biological tissues [28]. This will significantly simplify the early detection of diseases and improve patient treatment outcomes. Thus, EIM makes it possible to obtain an assessment of the composition and structure of muscles and surrounding tissues by measuring their resistive and capacitive properties, as well as to evaluate the muscle contraction force [11]. This opens up opportunities for wider clinical application of this method in assessing the condition of tissues, as well as in proportional biofeedback tasks.

Thus, currently, proportional force control of industrial/medical manipulators, as well as prostheses, orthoses and exoskeletons using EIM is a developing field [29]. The reason is that modern robotic devices controlled using the generally accepted and used method of electromyography (hereinafter EMG) [30] do not allow for precise force regulation [31], and control strategies implemented in commercially available devices still largely provide only binary, or all-or-nothing control [32,33]. The integration of EIM and EMG methods, which is being studied in global practice, makes it possible to create proportional control systems, where EMG encodes the user’s intention, and EIM isolates the amplitude component of the action being performed (in the absence of applied force) and the level of force (taking into account the start of force application) [34]. Furthermore, EIM allows for the evaluation of muscle parameters in the context of their condition and pathological changes [35], which is particularly important for personalizing the control of prostheses [31]. In rehabilitation robotics, particularly for exoskeletons designed to assist in walking or performing strength tasks, it is critical to accurately determine the intention of a person’s movement and synchronize the force of the robotic device with muscle activity. Furthermore, EIM can be used to control the contact of the device with the skin surface, preventing excessive or insufficient pressure of the electrodes to it, which affects the recorded signal, especially under dynamic recording conditions [20].

Thus, the increase in the number of publications over the past 10 years is due not only to the combination of advances in hardware and software for bioimpedance methods, but also to the expansion of the range of problems that can be solved using this method. EIM has proven itself as a method for screening and assessing the condition of muscle tissue [12]. Thus, in the field of rehabilitation and assessment of disease progression, EIM is used to quantitatively assess the recovery of muscle mass and function after a stroke [36], which allows physicians to objectively evaluate the effectiveness of therapy and promptly adjust the rehabilitation program. EIM can also be used to assess the progression of diseases such as Duchenne muscular dystrophy [37], amyotrophic lateral sclerosis [38], spinal muscular atrophy [39], etc.

However, EIM, despite the above-mentioned areas of implementation, has its limitations, since measurements obtained using surface electrodes provide generalized information about the area of interest and do not allow localization of changes at the level of individual layers or structures. The measured parameters are not direct physical properties of the muscle, but represent the integrated response of the entire structure within the recorded area. Changes in tissue parameters, such as muscle size, the relative position of the electrodes and muscles, the thickness of the skin-fat layer in the projection of the electrode systems and its change during contraction, make a significant contribution to the recorded electrical impedance signal. For the correct interpretation of EIM data, it is critical to understand which biological tissues contribute to the measured signal. For example, in [40], it was shown that the percentage of body fat in overweight and obese individuals significantly affects EIM signal parameters.

The key aspect determining the practical value of EIM is the establishment of a quantitative relationship between the measured impedance parameters and specific physiological conditions of muscles.

Currently, there are a number of review articles devoted to EIM, which consider the general principles of the method, its clinical applications and technical aspects of the process, in particular, those covered in [1,3,7,41]. In turn, among the most modern review publications, it is worth noting [42,43,44]. However, despite the studies devoted to the use of EIM in diagnostics, rehabilitation and biofeedback interfaces, how the methodological measurement parameters, individual anatomy, the muscle tissue conditions, the contributions of the skin-fat layer and the force tissue deformation during the generation of muscle effort affect the EIM signal generation and interpretation remains fragmented across individual studies and has not yet been systematically mapped. This gap is associated not only with the lack of individual empirical data, but also with the absence of a consistent explanation of which tissue and measurement factors determine changes in impedance parameters during morphofunctional changes in biological tissues. This review systematizes the existing evidence on the use of EIM to assess morphofunctional changes in muscle and surrounding tissues, and identifies the methodological and tissue-related factors that remain insufficiently characterized in diagnostic, rehabilitation, and biofeedback tasks.

Unlike existing reviews, this review focuses on the methodological foundations and practical aspects of EIM application, with an emphasis on the signal generation mechanisms. Based on the analysis, current problems and unresolved issues in this field are formulated and the most promising directions for future research are identified, which will help overcome existing methodological and technological limitations. Thus, this scoping review aims to answer the following questions: (1) What are the key methodological factors affecting EIM signal interpretation? (2) How does EIM correlate with muscle morphology and function? (3) What are the current limitations and future directions for EIM in clinical and bioengineering applications? These questions, together with the eligibility criteria, the search strategy and the data-charting form, were fixed in a review protocol agreed by all authors before the search was carried out; the protocol is reported in full in Section 2.

To achieve the stated objectives, a comprehensive analysis of scientific literature published between 2016 and 2025 was conducted using the international Scopus database. However, a limitation of the study is that the literature search was conducted only in Scopus and did not include additional databases such as PubMed and Web of Science. This approach was chosen given the broad interdisciplinary coverage of Scopus, which includes publications in the fields of medicine, biomedical engineering, neurosciences, rehabilitation, and related technical fields, consistent with the complex nature of the topic under consideration. Although the inclusion of PubMed and Web of Science could have expanded the pool of potentially relevant publications, the use of the Scopus database ensured representative coverage of the study area, avoided excessive duplication of records, and maintained the reproducibility of the search strategy through the use of uniform criteria for selection, filtering, and analysis of sources.

Key sources included research papers, review articles in English specifically devoted to EIM theory and practice. Background materials included studies using medical imaging (ultrasound, MRI, computed tomography (CT)) and functional diagnostics (dynamometry, electromyography) to verify structural and functional changes in skeletal muscles compared to EIM. Additional sources were consulted as needed to ensure completeness of presentation, substantiate methodological approaches, and interpret the results in the context of EIM application.

## 2. Materials and Methods (Scoping Review Approach)

In conducting this scoping review in order to cover studies aimed at using EIM to assess morphofunctional changes in biological tissues and biofeedback interfaces, the authors of this work were guided by the recommendations of The PRISMA extension for scoping reviews (PRISMA-ScR, 2018) [45].

This scoping review was designed, conducted and reported in accordance with the Preferred Reporting Items for Systematic Reviews and Meta-Analyses extension for Scoping Reviews (PRISMA-ScR) [45]. A completed PRISMA-ScR checklist, mapping each of the 22 reporting items to the corresponding section and page of this manuscript, is provided as Supplementary Material.

**Protocol and registration.** A review protocol was prepared and agreed by all authors before the literature search was executed. The protocol fixed the review questions, the population–concept–context framework, the eligibility criteria, the Scopus search strategy and the data-charting form; it is reported in full in Section 2.1, Section 2.2, Section 2.3 and Section 2.4, so that the review can be reproduced from this manuscript. The review was not registered in a public registry. PROSPERO does not accept registrations of scoping reviews, and no equivalent registration was undertaken in another registry; consequently, no registration number is available. No amendments were made to the protocol during the conduct of the review. The only addition was a small number of publications from 2026, which were used as supplementary background sources during revision and were deliberately kept outside the formal Scopus screening count (Section 2.3).

**Critical appraisal.** In accordance with PRISMA-ScR guidance, and consistent with the purpose of a scoping review, a formal critical appraisal or risk-of-bias assessment of the individual included sources was not performed. The objective of this review was to map the extent, range and nature of the available evidence on EIM signal generation and interpretation, rather than to estimate the effect of an intervention. Accordingly, no appraisal-based weighting, ranking or exclusion was applied in the synthesis. This is acknowledged among the limitations of the review in Section 4.2.

Funding of the individual included sources of evidence was not charted, as it was not relevant to the review questions, which concern measurement methodology and tissue physiology rather than intervention effects.

The literature search was conducted in December 2025 and updated in March 2026, including studies published from 2016 to 2025.

The screening process was primarily performed by one author, while two additional authors independently reviewed and validated the study selection and methodological approach; any disagreements were resolved through discussion to reach consensus.

### 2.1. Inclusion Criteria

For this review, inclusion criteria included research papers, review articles in English published within the last 10 years (2016–2025). This time period was chosen to analyze the current stage of EIM development, characterized by significant advances in the hardware implementation of measurement systems, multi-channel and multi-frequency methods, and the development of algorithms for processing and interpreting bioimpedance signals. This work reviewed and analyzed studies that used EIM for diagnostic, rehabilitation, and biofeedback purposes. Only studies that directly assessed the types of biological tissues studied, such as skeletal muscle and its conditions, skin, subcutaneous fat, and the contribution of these tissues to impedance signal generation, were selected for analysis.

Participant types: experimental/clinical studies involving humans (healthy volunteers, patients with specific diagnoses) or animals.

Data: availability of quantitative data on the relationship between impedance parameters and physiological (pathophysiological) tissue parameters (description of the relationship between impedance parameters (impedance modulus amplitude, phase angle, probing current frequency, active and reactive resistance) and tissue parameters (relative permittivity, specific electrical resistance, changes in internal geometry, etc.).

Use of standard measurement protocols: frequency ranges of 1 kHz–2 MHz, bipolar/tetrapolar technique.

### 2.2. Exclusion Criteria

The following were excluded from this review: studies using cell cultures; in vitro studies (using needle electrodes) with the exception of information for background references; studies solely devoted to whole-body bioimpedance analysis; studies without quantitative results that do not allow for establishing a relationship between EIM parameters and tissue condition; papers in languages other than English; duplicate data (duplicate publications on the same sample); studies containing measurements through clothing, bandages, gels with unknown conductivity, and measurements on non-intact skin (e.g., wounds, burns).

### 2.3. Data Sources

For this review, a literature search was conducted in Scopus using the following search query:

TITLE-ABS-KEY ((“electroimpedance” OR “bioimpedance” OR “electrical impedance” OR “electrical impedance spectroscopy” OR “electrical impedance tomography”) AND (“forearm muscles” OR “muscle assessment” OR “muscle function” OR “muscle activity” OR “muscle performance” OR “muscle contraction” OR “muscle diagnostics” OR “muscle strength” OR “muscle*”) AND (“morphofunctional changes” OR “muscle rehabilitation” OR “neuromuscular interface” OR “bioelectrical signals” OR “bioelectrical impedance” OR “functional assessment” OR “muscle control” OR “prosthetic control”)) AND (PUBYEAR > 2015 AND PUBYEAR < 2026) AND (LIMIT-TO (SUBJAREA, “MEDI”) OR LIMIT-TO (SUBJAREA, “NURS”) OR LIMIT-TO (SUBJAREA, “HEAL”) OR LIMIT-TO (SUBJAREA, “VETE”) OR LIMIT-TO (SUBJAREA, “NEUR”)) AND (LIMIT-TO (DOCTYPE, “ar”) OR LIMIT-TO (DOCTYPE, “re”)) AND (LIMIT-TO (LANGUAGE, “English”)). Publications from 2026 were added as supplementary sources during manuscript revision and were not included in the formal Scopus screening count.

To check for compliance with the selection criteria, a primary analysis of titles and abstracts was conducted. Each selected paper was analyzed for information on bibliographic characteristics (publication type, authors, year, and journal); study objectives; study design and methodology; study areas and tissue types examined; characteristics of EIM technique; results, conclusions, and stated limitations.

**Figure 1:**
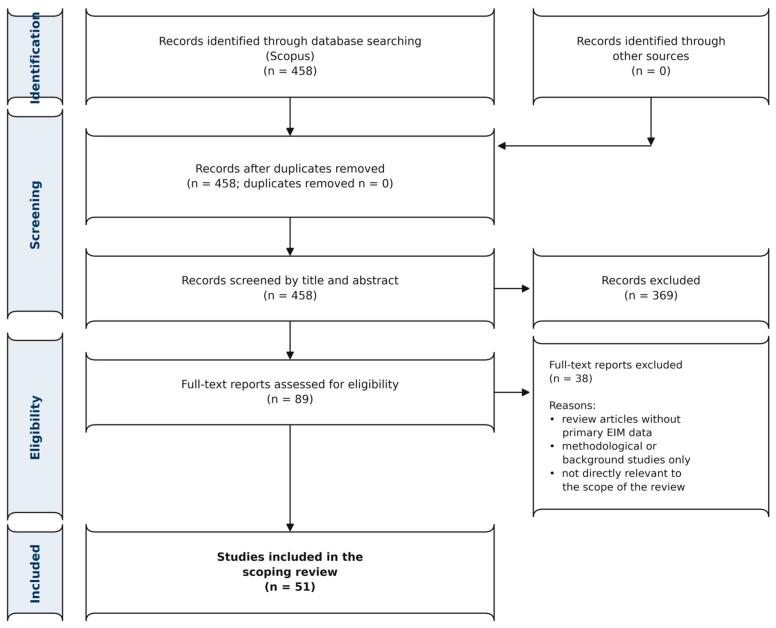
PRISMA-ScR flow diagram of the identification, screening and selection of sources of evidence included in the scoping review.

A total of 458 records were identified through the Scopus database search. No duplicates were detected in the dataset. After title and abstract screening, 369 records were excluded, resulting in 89 reports being retrieved for full-text assessment. Of these, 38 papers were excluded after full-text evaluation due to being review papers, methodological or background studies, or not directly relevant to the scope of the review. Ultimately, 51 studies met the inclusion criteria and were included in the final analysis. The identification, screening and selection process is summarized in Figure 1.

### 2.4. Research Records

Records were exported from the Scopus database to Zotero 7.0.32, a software for managing bibliographic data and associated research materials. The publication dataset was then screened for duplicate references.

Data extraction was performed using a predefined form. For each included paper, the following parameters were recorded: bibliographic characteristics of the publication, including paper type, authors, year of publication, and journal; study objective; design and methodology; field of EIM application, including diagnostics, rehabilitation, muscle function assessment, or biofeedback; study sample characteristics; tissue type and specific muscles or muscle groups examined; EIM technique parameters, including impedance parameters used, frequency or frequency range of the probing current, bipolar or tetrapolar electrode configuration, electrode material and placement, interelectrode distance, and measurement protocol details. Data on control or validation methods used for comparison with the EIM were also extracted, including dynamometry, electromyography, ultrasound, MRI, CT, and functional testing.

Quantitative results reflecting the relationship of EIM parameters with morphological and functional tissue characteristics, including correlation coefficients, regression model indicators, and the method’s sensitivity to changes in contractile force, fatigue, subcutaneous fat thickness, muscle cross-sectional area, adipose infiltration, atrophy, hypertrophy, and other morphofunctional changes, were separately recorded. This approach was chosen given that the interpretation of the EIM signal significantly depends on the configuration and placement of the electrodes, frequency range, anatomical features, and the measurement protocol.

For each paper, the authors’ main conclusions, stated study limitations, consideration/absence of consideration of the influence of skin and subcutaneous fat, and the presence of external validation or comparison with reference methods were also noted. This made it possible to systematize data not only by areas of EIM application, but also by methodological factors that determine the reproducibility and comparability of results between studies.

## 3. Results

### 3.1. Electroimpedance Techniques for Muscle Assessment

The choice of electrode system configuration is one of the fundamental aspects of EIM measurements, as it directly determines the sensitivity of the method to changes in specific tissue structures. The most common standard in modern practice remains the tetrapolar signal recording technique (4-electrode system), which minimizes the influence of resistance at the electrode-skin interface [46]. In contrast, the bipolar (2-electrode) system records the total resistance of the entire circuit, making the results sensitive to changes in the electrode-skin contact [44].

A key limitation of measurements using surface electrodes is that the impedance is a weighted average of all tissue layers through which the current passes, from the epidermis and subcutaneous fat to muscle fibers. Studies show that the contribution of the target muscle to the total signal when using standard surface electrodes can be less than 30% [8]. This makes data interpretation vulnerable to changes in the thickness of adipose tissue [47] or changes in the contact of the electrodes with the skin surface, which can significantly affect the result, masking or distorting true changes in the muscle [48]. Thus, for example, for the forearm muscles, for the purpose of recording signals in biofeedback systems, where the volume of muscle tissue is smaller and the anatomical complexity is higher, it is critical to consider the location of the electrode systems relative to the muscle active during the action, taking into account the myotendinous junction and the zones of greatest change in subcutaneous fat during contraction.

Also, the electrode shape and material significantly affect the current distribution and, consequently, the sensitivity of measurements to changes in tissue. The study [49] compared the effectiveness of round and rectangular electrodes in assessing forearm muscles. It was shown that rectangular electrodes, with a larger contact area, demonstrate a more pronounced dependence of the recorded signal on the thickness of subcutaneous fat. This means that in patients with variable body composition (e.g., with sarcopenic obesity or after injury), impedance changes measured with rectangular electrodes may be erroneously interpreted as muscle pathology, whereas in fact they reflect the characteristics of the subcutaneous fat. To minimize such artifacts, it is necessary to use calibration protocols that take into account the electrode configuration or apply multi-frequency analysis to isolate the contributions of different tissue structures [50].

The electrode material used also plays an important role in recording the EIM signal. Ag/AgCl provide a stable signal, but require the use of conductive gels [51], which can dry out or leak out and form a single conductive layer during contraction, and also cause impedance drift during long-term measurements. In the context of wearable devices, loss of contact or change in electrode-skin contact [52] can be mistakenly interpreted as a change in the user’s muscular conditions. A solution may be “dry” [53] or disposable electrodes; however, an actively developing direction in EIM electrode system configurations are flexible electrode matrices, which easily adapt to complex anatomical shapes [54,55], ensuring reliable contact with the skin during long-term wear. The geometry and interelectrode spacing of flexible electrode systems should be selected according to the user’s anatomy and the required probing depth [56].

In turn, the availability of a frequency range for the probing current during research is increasingly becoming a modern requirement for EIM systems. Different tissue structures and components exhibit different behavior at different frequencies of the alternating probing current, which allows for obtaining an impedance spectrum characteristic of a given tissue. EIM systems should allow measurements in the range from kilohertz to megahertz. For example, some modern interfaces are specifically designed to operate in the range from 1 kHz to 2 MHz (and higher), and the choice of a specific range depends on the research or diagnostic task. As noted in [57], insufficient range width or an incorrect choice of operating frequencies can lead to specific pathological changes being masked by general tissue impedance fluctuations. Therefore, it is critically important to validate the frequency protocol for a specific nosology or research task [58].

Thus, the selection of electrode configuration, material, geometry, and frequency protocol in EIM represents a comprehensive approach that determines the method’s sensitivity to the recorded morphofunctional changes in biological tissues. For accurate data interpretation, it is necessary to consider the influence of subcutaneous fat and, consequently, the location of electrode systems in the projection of target muscles, select the electrode configuration for a specific task, and use multi-frequency analysis to isolate the contributions of biological tissues.

### 3.2. Detection of Morphological and Functional Muscle Changes

For clarity, two distinct EIM measurement paradigms are considered throughout this review. In dynamic or time-resolved EIM, impedance is measured continuously or repeatedly during an ongoing muscle contraction or movement. The resulting within-trial changes reflect rapid alterations in tissue geometry, muscle fiber orientation, fluid distribution, and the relative positions of the tissues and electrodes. These measurements may be synchronized with dynamometry or EMG and used to investigate contraction-related changes or to develop force-estimation and biofeedback systems. In biofeedback applications, time-resolved EIM signals may serve as continuously updated control inputs for identifying movement phases, estimating relative changes in contraction force, adapting the assistance provided by a robotic device, and delivering immediate feedback to the user. However, such applications require appropriate individual calibration and control of measurement-related confounding factors. In contrast, a single-point or snapshot EIM assessment is obtained at a defined time point, typically under standardized resting conditions, and is used to characterize tissue composition and architecture or to compare muscles across individuals, clinical groups, or longitudinal visits.

Research conducted since 2016 demonstrates the high sensitivity of EIM to processes occurring in the muscle directly during contraction and recovery. These processes include changes in contractile force, as well as metabolic and structural changes in tissues, which are reflected in impedance characteristics.

The key parameters of EIM include: muscle active resistance (*R*), which reflects the resistance to current flow in extracellular and intracellular fluids; muscle reactance (*X*), which shows how cell membranes and fascia affect current flow; the phase angle, which is defined as follows:

(1) $$\theta =\arctan\;(\frac{X}{R}),$$

and the impedance modulus, which is defined as follows:

(2) $$\left|Z\right|=\;\sqrt{R^{2}+X^{2}}.$$

The authors of the paper [42] confirm that bioimpedance methods are sensitive to changes occurring after physical activity and may become a potential method for non-invasive quantitative assessment of changes in tissues caused by physical activity, fatigue, injury, and recovery. The use of EIM for assessing muscle strength is increasingly finding application, since changes in signal parameters may be associated with the superposition of myofibril excitation effects, displacement of intercellular fluid, an increase in the cross-sectional area of myofibrils (and the cross-sections of active muscles in general), and morphological changes (e.g., changes in skin-fat layer thickness, its displacement relative to electrode systems located on the skin surface). In turn, the authors of biological tissue study [28] showed that EIM parameters systematically change in response to isometric muscle contraction, both when maintaining the joint angle (in this case, changes in EIM parameters are mainly due to overcoming the force of gravity), and when actively pressing on a fixed lever (in this case, changes in EIM parameters are more pronounced and are directly related to the intensity of active contraction). With increasing force, an increase in active resistance and impedance modulus is observed, as well as a decrease in reactive resistance and phase angle. Thus, this study confirms that EIM is not just a marker of muscle structure, but also a sensitive tool for quantitatively assessing dynamic muscle activity and strength, provided that such factors as the type of contraction, joint angle and probing current frequency are taken into account.

In turn, in patients with stroke and hemiparesis, after a 40 min workout on a bicycle ergometer with functional electrical stimulation, statistically significant changes in EIM parameters were recorded, including those correlating with the results of clinical tests [2]. This study proves that EIM can objectively and non-invasively assess rapid intramuscular changes caused by the rehabilitation process.

Other studies confirm that EIM is sensitive to changes associated with muscle fatigue. Thus, in [22], it was shown that fatigue, assessed through the maximum voluntary contraction, correlated with changes in impedance (a trend towards a decrease in resistance R in the volunteer’s muscles from a condition of complete relaxation to the extreme degree of fatigue was shown, and also that at different load levels of 20%, 40%, 60% of maximum contraction). In turn, since continuous monitoring during the training process is extremely important for the prevention of muscle damage, this emphasizes the relevance of the potential EIM application in the detection of sports injuries [42,59], including in monitoring muscle fatigue conditions.

One of the most reliable and well-studied properties of EIM is its ability to reflect macroscopic changes in muscle tissue, such as changes in its cross-sectional area and composition, including atrophy, hypertrophy, adipose infiltration, and fibrosis. The authors [24] demonstrate that EIM can successfully monitor these processes. For example, an increase in the proportion of fat within the muscle should lead to an increase in the total impedance, which is supported by data [25] showing that EIM can effectively measure local fat content in muscles. Thus, another important systemic factor reflected in EIM parameters are morphofunctional changes caused by aging. In the context of age-related changes, EIM is a useful tool for screening sarcopenia [60], a syndrome characterized by a progressive loss of muscle mass and strength [61]. Phase angle values obtained using EIM have been shown to be a good marker in the assessment of sarcopenia, demonstrating a positive correlation with muscle mass and grip strength indices [62]. Below is a comparative Table 1 for some studies on EIM parameters and muscle strength indices (where the following abbreviations are used: R is active resistance; X is reactance; PhA is phase angle; MQ is muscle quality; hEIM is handheld electrical impedance myography; QMT is quantitative myometry; 6MWT is 6 min walk test; FSHD is facioscapulohumeral muscular dystrophy; FES is functional electrical stimulation.

The most significant biomarkers extracted from electrical impedance measurements are parameters that directly correlate with the morphology and physiological conditions of the muscle. A key such marker is the phase angle, which serves as a quantitative indicator of the ratio of reactive and active resistance. Its value directly depends on the structural and functional characteristics of the tissue under study. For example, in [62], volunteers with sarcopenia had significantly lower phase angle values compared to healthy volunteers; however, phase angle values have been shown to correlate with muscle strength and ultrasound-derived measures of muscle structure, supporting their potential use in the assessment of muscle condition [65]. Furthermore, since changes in muscle cells caused by contractions also lead to changes in muscle capacitance, monitoring changes in phase angle can become an additional reliable way to detect movement intention and record the strength of muscle contraction for proportional biofeedback tasks [66]. Below is Table 2, which summarizes some information from the sources found on the dependence of the key EIM parameters on various physiological and pathological conditions of muscle tissue (where the following abbreviations are used: R is active resistance; X is reactance; PhA is phase angle; MVC is maximum voluntary contraction; AR is anisotropy ratio).

### 3.3. Comparison of EIM with Control Methods

MRI, ultrasound, and CT have long been considered the reference method for assessing skeletal muscle morphology. However, the main limitation of these methods is their static nature, as they only allow for recording muscle and surrounding tissue conditions at a specific point in time but do not allow for observing its functional changes over time, for example, during contraction or fatigue. When recorded continuously or repeatedly during contraction, EIM can track time-resolved changes in the biophysical properties and geometry of the tissues within the measured region [42]. For example, to adjust the rehabilitation process after a stroke, it is important to also assess biomechanical changes unrelated to the nervous system [67]. Common morphological features found in stroke patients include decreased muscle cross-sectional area, mass, volume, increased intramuscular fat, changes in flexion angle and muscle bundle length, changes in which will ultimately lead to loss of muscle strength.

However, a comparison of EIM with the above-mentioned imaging methods shows that they do not so much compete as complement each other, creating the opportunity for a more complete picture of the muscle and surrounding tissue conditions. Thus, the findings of [68] indicate that EIM should not be used in isolation. Combining EIM with morphological (e.g., ultrasound/MRI/CT), mechanical (e.g., myotonometry, dynamometry) and functional methods for assessing muscle contractile properties, including the EMG method, increases the accuracy and informativeness of tissue parameter assessment [69]. In turn, the authors [63] showed that EIM correlates with both dual-energy X-ray absorptiometry data and with functional tissue parameters.

Due to the errors and operator dependence in measurements using ultrasound, particularly when using the free-hand method, where the key contribution is the operator’s influence, which can be eliminated by robotic ultrasound scanning in order to avoid compression of structures by the pressure of the ultrasound transducer exerted by the operator [70], MRI still remains the reference method for determining muscle morphology [71]. Thus, the addition of EIM to traditional methods of assessing tissue parameters (MRI, ultrasound) will provide unique information on the properties of target muscles at the micro level, inaccessible by visualization. At the same time, EIM demonstrates significant correlations with both morphological parameters according to MRI and with functional strength indicators, which ensures the validity of the method and makes it a cost-effective tool for non-invasive monitoring for diagnostic and proportional biofeedback tasks.

The authors [72] showed a significant reduction in the active resistance, reactance and phase angle in the region of the greater and lesser trochanter of the femur, recorded by the EIM method, which can be used in conjunction with imaging methods such as MRI and ultrasound. This is a valuable biomarker for non-invasive monitoring of skeletal muscle changes [73]. In study [64], ultrasound and EIM were evaluated as quantitative biomarkers in patients with Pompe disease, and the results demonstrated significant associations between EIM parameters, ultrasound-derived tissue characteristics, and measures of muscle strength. Pompe disease is a metabolic myopathy characterized by progressive skeletal muscle weakness [74].

The study [75], aimed at assessing muscle quality and percentage of fat, showed a weak correlation with other quantitative parameters used to assess muscle mass and adipose infiltration. However, an important fact is the consideration of the electrode system pressure (in this case, a portable EIM device) to the skin surface [21], which may not have been taken into account during the experiments. In turn, the study [76] demonstrated a relationship between EIM indicators and muscle structure measured using MRI. Below in Table 3, some parameters measured by EIM are presented and compared with MRI as a control method (where the following abbreviations are used: BF% is body fat percentage; MQ is muscle quality; FSHD is facioscapulohumeral muscular dystrophy).

Table 4 shows a comparison of EIM and ultrasound parameters in assessing the structural and functional characteristics of tissues (where the following abbreviations are used: SKfat is a local fat index output by the hEIM device; MQ is muscle quality; SUBfat is subcutaneous fat thickness; EIus is ultrasound echo intensity; ACSAQF is anatomical cross-sectional area of quadriceps femoris). The *p*-value for all parameters considered was *p* < 0.001; the authors’ study examined healthy young adults.

Thus, the EIM method does not compete with the reference methods of MRI, ultrasound (including CT, although this method is distinguished by the presence of radiation exposure), but complements them, providing a quantitative assessment of the biophysical properties of muscle tissue in real time. While imaging methods record static morphology, EIM allows for tracking functional changes in tissue directly during contraction (taking into account the force-torque parameters of the actions performed), while being distinguished by its speed and economic availability. The key limitations of the method remain its sensitivity to measurement conditions, which requires strict standardization of data collection protocols, including control of the pressure of the electrode systems, their geometry, interelectrode distance, and the number of measurement channels [19]. Thus, the strategy for the development of methods and research today involves the integration of imaging methods (MRI/ultrasound) for assessing anatomy and structure, as well as the EIM method for quantitatively assessing the functional conditions of muscle tissue in the same anatomical region. It is also necessary to take into account the dynamometry method for direct measurement of muscle strength and assessment of the functional result. This integrated approach will overcome the shortcomings of each individual method and create a holistic model of muscle and surrounding tissue conditions in real settings.

## 4. Discussion

The discussion of this review focuses on the gaps stated in the Introduction, namely, the lack of a clear understanding of how methodological measurement parameters, individual anatomy, muscle tissue condition, and the contribution of the skin-fat layer and tissue force deformation determine the generation and interpretation of the EIM signal. In this regard, the results of this review are discussed not only in terms of EIM application areas, but also in terms of factors limiting the reproducibility, comparability, and physiological interpretation of impedance parameters. It is important to note that additional sources not included in the main analysis, but relevant for the discussion of the mechanisms, are used to interpret the obtained results. EIM is a relatively new but rapidly developing non-invasive method for assessing muscle tissue condition. Unlike EMG, which records electrical potentials arising from muscle fiber activation, EIM focuses on measuring the physical properties of the tissue itself, i.e., its composition and architecture.

Unlike EMG, which records endogenous electrical potentials generated during muscle-fiber activation, EIM applies a weak, high-frequency current without inducing myofiber or neuronal action potentials and characterizes passive volume-conduction properties related to tissue composition and architecture [1,41]. Thus, EMG and EIM provide complementary information about muscle activation and tissue state.

In recent years, a number of review articles have been published on EIM, covering both the fundamental principles of the method and its clinical and engineering applications. Thus, the studies [1,7,41] present the key principles of EIM and its application in neuromuscular diseases, while [3] summarize the use of the method in conditions of physical activity and in healthy subjects. More recent reviews such as [42,43] focus on technological developments, wearable systems and the integration of EIM with other bioimpedance approaches. In [44], the authors consider bioimpedance in the context of physical activity monitoring. In contrast to these studies, the review we present focuses on an in-depth interpretation of EIM as a method for assessing the morphofunctional parameters of tissues for rehabilitation problems, diagnostics and the potential application of the method for solving problems of anthropomorphic biofeedback, taking into account the force-moment characteristics of the actions performed, changes in the architecture of soft tissues during contraction, as well as the integration of EIM data with the results of other imaging and functional diagnostic methods, thereby expanding the existing understanding of the capabilities of this approach.

### 4.1. Integrating EIM with Other Modalities

One of the key findings of modern bionic control research is the fundamental limitations of single sensory modalities. Surface EMG, a traditional biofeedback method, suffers from recorded signal crosstalk and non-stationarity, which reduces the reliability of control signal strength prediction models, especially with prolonged use or changes in muscle condition [77]. Unlike EMG, EIM is a method sensitive to tissue morphological changes. Integrating EIM and EMG data helps overcome the shortcomings of each method. A key advantage of this approach is the system’s ability to distinguish between movement phases and force levels. For example, the study [78] demonstrated that a multimodal system integrating EMG and EIM outperforms unimodal analogs in predicting the force of a performed action. The authors showed that EIM is more sensitive to changes in static force than surface EMG, especially at low levels of muscle activation. The approach of integrating the EIM and EMG methods makes it possible to obtain, for example, data on the stages of the performed action (e.g., extension/flexion of the wrist to an angle in the absence of applied force) using EIM with the placement of electrode systems in the projection of the muscles active during the performance of the action, as well as on the increase in contractile force using surface EMG. This, in turn, opens up new possibilities for the comprehensive assessment of muscle function, particularly for proportional biofeedback tasks.

It is important to note that a promising strategy for creating reliable, adaptive, and durable control and condition assessment systems is not the replacement of one technology with another, but their integration. For example, a system built on multimodal sensors, such as bioimpedance, EMG, and myotonography, will allow for the extraction of the most comprehensive information about the user’s motor intention [79], as well as the real-time assessment of the neuromuscular system conditions, since the key advantage of hybrid systems lies in their ability to compensate for the shortcomings of each individual modality. For example, EIM data can be used to assess muscle condition and calibrate the entire control system. This approach not only improves accuracy but also makes the system more fault-tolerant.

Currently, research is being conducted aimed at integrating electrical impedance, electromyography, and force myography methods for biofeedback tasks [80]. Another approach proposes using impedance changes in a specific part of the body to assess kinematics (e.g., the flexion-extension angle at the wrist), which opens up the possibility of creating new types of bionic prostheses [81]. It is important to note that, in addition to EMG, other sensory modalities can also serve as additional methods to EIM, such as optical myography [82] and inertial sensors [83]. They are also independent methods for recording neuromuscular activity, which in themselves can complement or replace EMG. EIM can also provide information necessary for calibrating these systems or for adjusting the operation of biosensor systems, for example, for assessing displacements caused by movement.

The role of ultrasound and MRI in the context of biofeedback systems is to validate and calibrate the EIM systems. To trust the data obtained from a portable EIM device, it is necessary to establish its correlation with reference methods, which remains a challenge for researchers today. By comparing changes in impedance biomarkers with changes in morphological parameters measured using ultrasound/MRI, it is possible to create a mathematical model that will translate real-time available EIM parameters into interpretable clinical units or biofeedback system parameters.

Thus, the most promising strategy for creating reliable and adaptive systems for assessing tissue morphofunctional parameters and biofeedback systems is not the mutual replacement of biosignal recording methods, but their integration. Combining electrical impedance data with methods from other modalities allows for the creation of a control interface capable of not only determining the user’s intention to perform a movement [84], but also assessing the neuromuscular system conditions and determining the muscle contractile force [28].

### 4.2. Limited Data Availability and Methodological Gaps

Despite the enormous potential of EIM, its widespread adoption in clinical practice and in commercial biofeedback systems is still hindered by a number of limitations in the fundamental aspects of the signal measurement process itself and the mechanisms by which it is generated, as well as the technical features of the hardware and software of existing commercially available systems. One of the key issues remains the quality of the electrode-skin contact [85], as well as the dependence of measurements on individual anatomical and physiological characteristics of a person [3,41]. Processes associated with muscle fatigue are also reflected in impedance signals, but their interpretation remains ambiguous and requires additional validation.

A significant limitation remains the insufficient understanding of EIM signal generation mechanisms. Specifically, muscle contraction alters the geometry and relative positions of the tissues within the measurement region. Ultrasound observations have shown that, under low electrode-system pressure, contraction may also be accompanied by local thinning or displacement of the subcutaneous fat layer, which can affect the recorded impedance signal [20]. However, the contribution of individual components (muscle tissue, subcutaneous fat) to the generation of the final signal remains insufficiently quantified, complicating data interpretation, especially under dynamic conditions.

The limitations of this review should also be noted. Differences in the configuration of electrode systems, frequency ranges, and measurement protocols in the reviewed studies complicate direct correlation of the results of various studies and limit the possibility of a rigorous comparison. Furthermore, some modern studies focus on the use of signal processing methods (in particular, using machine learning), but do not consider the signal generation mechanisms or the relationship with morphofunctional tissue parameters. Thus, taking into account the identified gaps, it is possible to formulate a number of practical recommendations for future research. Standardization of EIM measurement protocols for each specific research or diagnostic task is necessary. An important area is the development of models describing the contribution of various tissues to the impedance signal generation, especially under conditions of changing muscle load. A relevant area of research is conducting studies with the simultaneous use of EIM and imaging methods (MRI, ultrasound) for a more accurate interpretation of impedance parameters with an assessment of the contribution of changing biological tissue parameters to the recorded EIM signal. Finally, there is a need for expanded clinical and applied research involving various groups of subjects, as well as validation of approaches on large samples to improve the method’s reliability and reproducibility, and accelerate EIM implementation into practice.

Moreover, the suitability of EIM for frequent at-home self-assessment may facilitate the detection of gradual disease- or treatment-related changes that could be missed by infrequent MRI or CT examinations [86]. A recent cross-sectional study demonstrated that machine learning applied to multifrequency EIM data can improve the screening of neuromuscular disorders. Using measurements from nine muscles in 119 adults and 111 children, the authors reported participant-level accuracies of up to 84.0% in adults and 92.8% in children, while regression models also showed potential for predicting muscle strength. Although the study primarily evaluated diagnostic screening, its findings also support the broader use of EIM for repeated assessment of disease progression and treatment response, including measurements performed in the clinic or potentially at home. However, these longitudinal applications require further validation in prospective studies [87].

One of the limitations of this scoping review is that the literature search was conducted exclusively in the Scopus database, without additional inclusion of PubMed, Web of Science, and other databases. This could have resulted in incomplete coverage of individual publications indexed exclusively in other bibliographic databases and, consequently, the potential exclusion of some relevant studies. However, the choice of the Scopus database was due to its broad interdisciplinary coverage, which is particularly important for the topic of EIM, which lies at the intersection of medicine, biomedical engineering, neuroscience, physiology, rehabilitation, and biofeedback technologies. Furthermore, the use of a single database ensured uniformity of the search strategy, reproducibility of selection, and a reduced risk of uncontrolled duplication of records. Thus, despite this limitation, the Scopus abstract database provided a representative array of publications sufficient to identify the main directions, methodological features, and gaps in the study area.

Animal models remain important for the validation of EIM. Preclinical studies allow impedance measurements to be combined with controlled disease or aging models, direct assessment of muscle contractile function, and subsequent histological analysis. This approach helps clarify how specific tissue changes affect EIM parameters. However, a comprehensive analysis of animal-model studies is beyond the scope of the present review and warrants a separate dedicated review.

The results of this review help expand understanding the relationship between methodological and morphofunctional factors, on the one hand, and the EIM signal parameters, on the other. Thus, the review advances the current understanding of the EIM signal generation mechanisms outlined in the Introduction, demonstrating that the interpretation of this signal must take into account not only the target muscle conditions, but also the electrode system configuration, the frequency range, the skin-fat layer thickness and displacement, and changes in tissue geometry, particularly those of the muscles active during contraction. At the same time, there remains a need for further research aimed at quantitatively isolating the contributions of individual biological tissues to the recorded signal.

## 5. Conclusions

An analysis of studies conducted from 2016 to 2025 demonstrates that EIM is a rapidly developing, promising method for the comprehensive assessment of both rapid and slow morphofunctional changes in muscles. However, widespread clinical implementation and use in biofeedback and diagnostic systems are hampered by the complexity of EIM signal interpretation, due to its multicomponent nature and dependence on the internal geometry of tissues. A key limitation remains the insufficient understanding of the signal generation mechanisms, specifically the lack of reliable methods for isolating the contribution of the target muscle and subcutaneous fat to the recorded signal. This review demonstrates that EIM signal interpretation significantly depends on a number of methodological factors, including electrode configuration and placement, frequency range selection, anatomical features, and the measurement protocol, which directly impacts the reproducibility and comparability of results. The review demonstrated a relationship between EIM parameters and the morphological and morphofunctional characteristics of muscle tissue, including the ability to reflect tissue changes caused by changes in muscle contraction intensity. This confirms the potential of EIM as a noninvasive diagnostic tool and a method of proportional biofeedback.

To overcome the existing limitations of the method, the integration of experimental data with finite element modeling of current propagation in multilayer biological media, taking into account the real geometry, is a necessary vector of future research. A priority area is also conducting additional studies, including a comparison of EIM with reference imaging methods (e.g., MRI, ultrasound) to quantitatively assess the contribution of various biological tissues in order to determine the location and configuration of electrode systems. Further development of EIM is impossible without the creation of hybrid multimodal systems combining EIM with electromyography and dynamometry to improve the accuracy of decoding the user’s motor intentions. In turn, the development of adaptive signal processing algorithms and flexible wearable sensor matrices will minimize the influence of external artifacts and ensure the stability of measurements in real conditions. Active implementation of machine learning methods in the evaluation of EIM signals [88] will help overcome a number of existing limitations; however, this step should not be taken without a thorough study of the signal generation mechanisms, which can entail serious consequences, from the misinterpretation of clinical data to errors in biofeedback commands. Only a deeper understanding of the biophysical basis of signal generation and the transition to personalized tissue models will ensure the transformation of EIM from a research tool into a reliable clinical tool for rehabilitation and biofeedback tasks.

## Figures and Tables

**Table 1:** Correlation of EIM parameters with recorded force parameters.

Functional Parameter	EIM Parameter	Strength Assessment Method	Correlation Coefficient (r)	*p*-Value	Population
Knee extensor strength	R, 200 kHz	Biodex, normalized peak torque (knee extension)	−0.57	*p* = 0.01	Older adults, anterior thigh muscle group [63]
Knee extensor strength	PhA, 200 kHz	Biodex, normalized peak torque (knee extension)	−0.54	*p* = 0.01	Older adults, anterior thigh muscle group [63]
Knee extensor strength	R, 200 kHz	Biodex, normalized peak torque (knee extension)	−0.53	*p* = 0.01	Older adults, anterior thigh muscle group [63]
Knee extensor strength	PhA, 200 kHz	Biodex, normalized peak torque (knee extension)	−0.51	*p* = 0.02	Older adults, anterior thigh muscle group [63]
Knee extensor strength	Muscle fat %, hEIM	hEIM vs. normalized peak torque	−0.49	*p* = 0.009	Older adults, anterior thigh muscle group [63]
Functional mobility	MQ, deltoid	6MWT	0.62	*p* = 0.0047	Late-onset Pompe-disease [64]
Functional activity of lower extremities	MQ, deltoid	30 s chair stand test	0.78	*p* = 0.0015	Late-onset Pompe-disease [64]
Grip strength	Body fat % above forearm flexors	Handgrip dynamometry	−0.59	*p* = 0.0217	Late-onset Pompe-disease [64]
Walking distance	Reactance, 50 kHz, leg summary score	6MWT	0.74	*p* < 0.0001	FSHD [35]
Knee extensor strength	Reactance, 50 kHz, right vastus lateralis	QMT, right knee extensor percent predicted	0.49	*p* = 0.003	FSHD [35]
Elbow flexor strength	Reactance, 50 kHz, arm summary score	QMT, right elbow flexor percent predicted	0.57	*p* = 0.001	FSHD [35]

**Table 2:** Dependence of EIM parameters on muscle tissue conditions and morphofunctional changes.

Condition/Change	EIM Parameter	Nature of Change
Isometric biceps brachii contraction, 60% MVC	R	Increase [11]
Isometric biceps brachii contraction, MVC	R	Increase [11]
Sustained fatigue at 60% MVC to failure	R	Decrease [22]
Dynamic contraction to exhaustion	R	Decrease [22]
Chronic stroke, paretic biceps brachii	X, PhA, AR(R), AR(X)	Difference between groups; decrease on paretic side [36]
Chronic stroke, immediate effect of FES-assisted cycling in tibialis anterior and medial gastrocnemius	X, PhA	Increase [2]
Chronic stroke, immediate effect of FES-assisted cycling	R	Increase [2]

**Table 3:** Comparative analysis of EIM and MRI data.

Measured Parameter	EIM Parameter	MRI Parameter	Correlation Coefficient (ρ/r)
Structural severity of muscle damage in FSHD	Reactance, 50 kHz	MRI T1 muscle score	ρ = −0.71 [76]
Adipose infiltration in FSHD	Reactance, 50 kHz	MRI Dixon fat fraction	ρ = −0.74 [76]
Adipose replacement in muscle	Phase angle at multiple frequencies	MRI fat fraction/fat replacement	ρ = −0.53–0.73 [69]
Subcutaneous fat layer thickness	Resistance	MRI subcutaneous fat width	ρ = 0.65–0.88 [69]
Adipose infiltration of lumbar muscles	BF% (Skulpt hEIM)	Goutallier score	r = 0.26 [75]
Lumbar muscle quality	MQ (Skulpt hEIM)	Goutallier score	r = −0.22 [75]
Adipose infiltration of lumbar muscles in patients 18–40 years old	BF% (Skulpt hEIM)	Goutallier score	r = 0.485 [75]
Lumbar muscle quality in patients 18–40 years old	MQ (Skulpt hEIM)	Goutallier score	r = −0.401 [75]

**Table 4:** Comparative analysis of EIM and ultrasound data [25].

Measured Parameter	EIM Parameter	Ultrasound Parameter	Correlation Coefficient (r)
Localized fat content	SKfat	SUBfat	0.88
Localized fat content	SKfat	EIus	0.64
Muscle quality	MQ	EIus	−0.66
Muscle size	MQ	ACSAQF	0.37
Combined muscle quality/subcutaneous fat index	MQ	EIus/SUBfat	0.37
Combined muscle area/subcutaneous fat index	MQ	ACSAQF/SUBfat	0.81

## Data Availability

The original contributions presented in this study are included in the article. Further inquiries can be directed to the corresponding author.

## Supplementary Materials

The following supporting information can be downloaded at: https://www.mdpi.com/article/10.3390/s26154926/s1, PRISMA-ScR checklist.

## Abbreviations

The following abbreviations are used in this manuscript:

EIM	electrical impedance myography
EMG	electromyography
MRI	magnetic resonance imaging
CT	computed tomography
PRISMA	Preferred Reporting Items for Systematic Reviews and Meta-Analyses
PRISMA-ScR	PRISMA extension for Scoping Reviews
BIA	bioimpedance analysis
BIVA	bioelectrical impedance vector analysis
EIT	electrical impedance tomography
EI	electrical impedance
FMG	force myography
R	active resistance
X	reactance
PhA	phase angle
MQ	muscle quality
MVC	maximum voluntary contraction
AR	anisotropy ratio
QMT	quantitative myometry
6MWT	6 min walk test
FES	functional electrical stimulation
FSHD	facioscapulohumeral muscular dystrophy
BF%	body fat percentage
SKfat	local fat index
SUBfat	subcutaneous fat thickness
EIus	ultrasound echo intensity
ACSAQF	anatomical cross-sectional area of quadriceps femoris
ALS	amyotrophic lateral sclerosis
DMD	Duchenne muscular dystrophy
SMA	spinal muscular atrophy

## References

[1] Sanchez B., Rutkove S.B. (2017). Electrical Impedance Myography and Its Applications in Neuromuscular Disorders. Neurotherapeutics.

[2] Li L., Hu C., Leung K.W., Tong R.K. (2022). Immediate Effects of Functional Electrical Stimulation-Assisted Cycling on the Paretic Muscles of Patients with Hemiparesis after Stroke: Evidence from Electrical Impedance Myography. Front. Aging Neurosci..

[3] Cebrián-Ponce Á., Irurtia A., Carrasco-Marginet M., Saco-Ledo G., Girabent-Farrés M., Castizo-Olier J. (2021). Electrical Impedance Myography in Health and Physical Exercise: A Systematic Review and Future Perspectives. Front. Physiol..

[4] Xu P., Yang X., Ma W., He W., Vasić Ž.L., Cifrek M., Gao Y. (2023). A Study of Handgrip Force Prediction Scheme Based on Electrical Impedance Myography. IEEE J. Electromagn. RF Microw. Med. Biol..

[5] Nescolarde L., Talluri A., Yanguas J., Lukaski H. (2023). Phase Angle in Localized Bioimpedance Measurements to Assess and Monitor Muscle Injury. Rev. Endocr. Metab. Disord..

[6] Ward L.C., Brantlov S. (2023). Bioimpedance Basics and Phase Angle Fundamentals. Rev. Endocr. Metab. Disord..

[7] Rutkove S.B., Sanchez B. (2019). Electrical Impedance Methods in Neuromuscular Assessment: An Overview. Cold Spring Harb. Perspect. Med..

[8] Rutkove S.B., Pacheck A., Sanchez B. (2017). Sensitivity Distribution Simulations of Surface Electrode Configurations for Electrical Impedance Myography. Muscle Nerve.

[9] Baidillah M.R., Riyanto R., Busono P., Karim S., Febryarto R., Astasari A., Sangaji D., Taruno W.P. (2024). Electrical Impedance Spectroscopy for Skin Layer Assessment: A Scoping Review of Electrode Design, Measurement Methods, and Post-Processing Techniques. Measurement.

[10] Sanchez B., Pacheck A., Rutkove S.B. (2016). Guidelines to Electrode Positioning for Human and Animal Electrical Impedance Myography Research. Sci. Rep..

[11] Li L., Shin H., Li X., Li S., Zhou P. (2016). Localized Electrical Impedance Myography of the Biceps Brachii Muscle during Different Levels of Isometric Contraction and Fatigue. Sensors.

[12] Clark B.C., Rutkove S., Lupton E.C., Padilla C.J., Arnold W.D. (2021). Potential Utility of Electrical Impedance Myography in Evaluating Age-Related Skeletal Muscle Function Deficits. Front. Physiol..

[13] Yu Y., Kalra A.M., Anand G., Lowe A. (2023). A Pilot Study Examining the Dielectric Response of Human Forearm Tissues. Biosensors.

[14] Briko A., Kapravchuk V., Kobelev A., Tikhomirov A., Hammoud A., Al-Harosh M., Leonhardt S., Ngo C., Gulyaev Y., Shchukin S. (2021). Determination of the Geometric Parameters of Electrode Systems for Electrical Impedance Myography: A Preliminary Study. Sensors.

[15] Ge X., Zhang L., Xiang G., Hu Y., Lun D. (2020). Cross-Sectional Area Measurement Techniques of Soft Tissue: A Literature Review. Orthop. Surg..

[16] Yu F., Zeng L., Pan D., Sui X., Tang J. (2020). Evaluating the Accuracy of Hand Models Obtained from Two 3D Scanning Techniques. Sci. Rep..

[17] Silva R., Silva B., Fernandes C., Morouço P., Alves N., Veloso A. (2024). A Review on 3D Scanners Studies for Producing Customized Orthoses. Sensors.

[18] Yu Y., Anand G., Lowe A., Zhang H., Kalra A. (2022). Towards Estimating Arterial Diameter Using Bioimpedance Spectroscopy: A Computational Simulation and Tissue Phantom Analysis. Sensors.

[19] Kapravchuk V., Briko A., Kobelev A., Hammoud A., Shchukin S. (2024). An Approach to Using Electrical Impedance Myography Signal Sensors to Assess Morphofunctional Changes in Tissue during Muscle Contraction. Biosensors.

[20] Kobelev A.V., Briko A.N., Kapravchuk V.V., Popova V., Goidina T., Maslennikova E., Shchukin S. (2022). Investigation of Pressing the Electrode System on the EIM Signals and Morphological Forearm Tissue Changes during Muscle Contraction. Biomed. Radioelectron..

[21] Briko A., Kapravchuk V., Selutina S., Shchukin S., Gulyaev Y., Leonhardt S. (2021). Amplitude Parameters of Electrical Impedance Myography with Different Pressure of the Electrode System Research. Proceedings of the 2021 Ural Symposium on Biomedical Engineering, Radioelectronics and Information Technology (USBEREIT), Yekaterinburg, Russia, 13–14 May 2021.

[22] Huang L.K., Huang L.N., Gao Y.M., Vasić Ž.L., Cifrek M., Du M. (2020). Electrical Impedance Myography Applied to Monitoring of Muscle Fatigue during Dynamic Contractions. IEEE Access.

[23] Nagy J.A., Kapur K., Taylor R.S., Sanchez B., Rutkove S.B. (2018). Electrical Impedance Myography as a Biomarker of Myostatin Inhibition with ActRIIB-mFc: A Study in Wild-Type Mice. Future Sci. OA.

[24] Rutkove S.B., Callegari S., Concepcion H., Mourey T., Widrick J., Nagy J.A., Nath A.K. (2023). Electrical Impedance Myography Detects Age-Related Skeletal Muscle Atrophy in Adult Zebrafish. Sci. Rep..

[25] Longo S., Coratella G., Rampichini S., Borrelli M., Scurati R., Limonta E., Cè E., Esposito F. (2021). Local Fat Content and Muscle Quality Measured by a New Electrical Impedance Myography Device: Correlations with Ultrasound Variables. Eur. J. Sport Sci..

[26] Mahato N.K., Cobb B.S., Rutkove S.B., Arnold W.D., Clark B.C. (2025). Multi-Frequency Electrical Impedance Myography as a Predictor of Muscle Function in Older Adults. Muscle Nerve.

[27] Carson R.G. (2018). Get a Grip: Individual Variations in Grip Strength Are a Marker of Brain Health. Neurobiol. Aging.

[28] Hülkenberg A.C., Strotmann N., Mokni A., Greulich L., Laurentius T., Bollheimer C., Rutkove S., Freeborn T., Leonhardt S. (2025). Electrical Impedance Myography Quantifies Holding and Pushing during Isometric Muscle Contractions. IEEE Trans. Neural Syst. Rehabil. Eng..

[29] Li J., Niu B., Wu K., Xiao J., Liu X., Wang Y. (2025). Bioelectronic Sensors for Neuromuscular Perception in Human-Machine Interfaces. Adv. Robot. Res..

[30] Chadwell A., Kenney L., Thies S., Galpin A., Head J. (2016). The Reality of Myoelectric Prostheses: Understanding What Makes These Devices Difficult for Some Users to Control. Front. Neurorobotics.

[31] Schulz A., Egle F., Osswald M., Del Vecchio A., Castellini C. (2025). Towards Unsupervised Incremental and Proportional Myocontrol Based on Higher-Density Surface Electromyography. Proceedings of the 2025 International Conference on Rehabilitation Robotics (ICORR), Chicago, IL, USA, 12–16 May 2025.

[32] Thomson C.J., George J.A. (2022). Proportional Electromyographic Control of a Bionic Arm in a Participant with Chronic Hemiparesis, Muscle Spasticity, and Impaired Range of Motion: A Case Study. Myoelectric Controls Symposium.

[33] Fu J., Choudhury R., Hosseini S.M., Simpson R., Park J.-H. (2022). Myoelectric Control Systems for Upper Limb Wearable Robotic Exoskeletons and Exosuits—A Systematic Review. Sensors.

[34] Ngo C., Munoz C., Lueken M., Hülkenberg A., Bollheimer C., Briko A., Kobelev A., Shchukin S., Leonhardt S. (2022). A Wearable, Multi-Frequency Device to Measure Muscle Activity Combining Simultaneous Electromyography and Electrical Impedance Myography. Sensors.

[35] Statland J.M., Heatwole C., Eichinger K., Dilek N., Martens W.B., Tawil R. (2016). Electrical Impedance Myography in Facioscapulohumeral Muscular Dystrophy: EIM in FSHD. Muscle Nerve.

[36] Li X., Li L., Shin H., Li S., Zhou P. (2017). Electrical Impedance Myography for Evaluating Paretic Muscle Changes after Stroke. IEEE Trans. Neural Syst. Rehabil. Eng..

[37] Rutkove S.B., Kapur K., Zaidman C.M., Wu J.S., Pasternak A., Madabusi L., Yim S., Pacheck A., Szelag H., Harrington T. (2017). Electrical Impedance Myography for Assessment of Duchenne Muscular Dystrophy. Ann. Neurol..

[38] Shefner J.M., Rutkove S.B., Caress J.B., Benatar M., David W.S., Cartwright M.S., Macklin E.A., Bohorquez J.L. (2018). Reducing Sample Size Requirements for Future ALS Clinical Trials with a Dedicated Electrical Impedance Myography System. Amyotroph. Lateral Scler. Front. Degener..

[39] Sonbas Cobb B., Kolb S.J., Rutkove S.B. (2024). Machine Learning-Enhanced Electrical Impedance Myography to Diagnose and Track Spinal Muscular Atrophy Progression. Physiol. Meas..

[40] van Rassel C.R., Bewski N.A., O’Loughlin E.K., Wright A., Scheel D.P., Puig L., Kakinami L. (2018). Validity of Electrical Impedance Myography to Estimate Percent Body Fat: Comparison to Bio-Electrical Impedance and Dual-Energy X-Ray Absorptiometry. J. Sports Med. Phys. Fit..

[41] Sanchez B., Martinsen O.G., Freeborn T.J., Furse C.M. (2021). Electrical Impedance Myography: A Critical Review and Outlook. Clin. Neurophysiol..

[42] Xu P., Zhou J., Chen Z.D., Yang X., Yan H., Vasić Ž.L., Cifrek M., Pun S.H., Vai M.I., Gao Y. (2024). Advancements and Challenges in Electrical Impedance Myography (EIM): A Comprehensive Overview of Technology Development, Applications in Sports Health, and Future Directions. IEEE J. Microw..

[43] Youssef Baby L., Bedran R.S., Doumit A., El Hassan R.H., Maalouf N. (2024). Past, Present, and Future of Electrical Impedance Tomography and Myography for Medical Applications: A Scoping Review. Front. Bioeng. Biotechnol..

[44] Jacobs I., Lowe A., Garcia L., Zhang H. (2026). Advancing Physical Activity Monitoring through Bioimpedance Measurement: A Review. Prog. Biomed. Eng..

[45] Tricco A.C., Lillie E., Zarin W., O’Brien K.K., Colquhoun H., Levac D., Moher D., Peters M.D.J., Horsley T., Weeks L. (2018). PRISMA Extension for Scoping Reviews (PRISMA-ScR): Checklist and Explanation. Ann. Intern. Med..

[46] Kassanos P. (2021). Bioimpedance Sensors: A Tutorial. IEEE Sens. J..

[47] Yao T., Wu Y., Jiang D., Bayford R., Demosthenous A. (2025). Forearm Motion and Hand Grasp Prediction Based on Target Muscle Bioimpedance for Human–Machine Interaction. IEEE Trans. Neural Syst. Rehabil. Eng..

[48] Wang J.N., Zhou H.Y., Gao Y.M., Yang J.J., Lučev Vasić Ž., Cifrek M., Du M. (2020). Optimization of the Electrode Configuration of Electrical Impedance Myography for Wearable Application. Automatika.

[49] Ahad M.A., Baidya S., Tarek M.N. (2023). Comparison of Circular and Rectangular-Shaped Electrodes for Electrical Impedance Myography Measurements on Human Upper Arms. Micromachines.

[50] Ballarin G., Valerio G., Alicante P., Di Vincenzo O., Monfrecola F., Scalfi L. (2024). Could BIA-Derived Phase Angle Predict Health-Related Musculoskeletal Fitness? A Cross-Sectional Study in Young Adults. Nutrition.

[51] Murphy B.B., Scheid B.H., Hendricks Q., Apollo N.V., Litt B., Vitale F. (2021). Time Evolution of the Skin–Electrode Interface Impedance under Different Skin Treatments. Sensors.

[52] Matsukawa R., Miyamoto A., Yokota T., Someya T. (2020). Skin Impedance Measurements with Nanomesh Electrodes for Monitoring Skin Hydration. Adv. Healthc. Mater..

[53] Yamagami M., Peters K.M., Milovanovic I., Kuang I., Yang Z., Lu N., Steele K.M. (2018). Assessment of Dry Epidermal Electrodes for Long-Term Electromyography Measurements. Sensors.

[54] Wu H., Long F., Mao H., Moon J., Zhu J., Luo Y. (2025). BandEI: A Flexible Electrical Impedance Sensing Bandage for Deep Muscles and Tendons. Proceedings of the 38th Annual ACM Symposium on User Interface Software and Technology, Busan, Republic of Korea, 28 September–1 October 2025.

[55] Murphy B.B., Mulcahey P.J., Driscoll N., Richardson A.G., Robbins G.T., Apollo N.V., Maleski K., Lucas T.H., Gogotsi Y., Dillingham T. (2023). A Gel-Free Ti3C2Tx-Based Electrode Array for High-Density, High-Resolution Surface Electromyography. MXenes.

[56] Kobelev A.V., Shchukin S.I., Leonhardt S. (2019). Application of Tetrapolar Electrode Systems in Electrical Impedance Measurements. Biomed. Eng..

[57] Schrunder A.D.F., Huang Y.-K., Rodriguez S., Rusu A. (2024). A Bioimpedance Spectroscopy Interface for EIM Based on IF-Sampling and Pseudo 2-Path SC Bandpass ΔΣ ADC. IEEE Trans. Biomed. Circuits Syst..

[58] Asano Y., Yoshida T., Tsunoda K., Yokoyama K., Watanabe Y., Yoshinaka Y., Okura T., Kimura M., Yamada Y. (2025). Segmental Validation of the 50-kHz Phase Angle as a Valid, Practical Proxy That Closely Approximates the Characteristic-Frequency Phase Angle and Associations with Muscle Function and Body Water: A Cross-Sectional Study. Clin. Nutr. ESPEN.

[59] Liu Y., Xu X., Yang A. (2025). Bioelectrical Impedance Vector Analysis (BIVA) in Sports Science: Applications, Insights and Future Directions. Int. J. Electrochem. Sci..

[60] Arnold W.D., Taylor R.S., Li J., Nagy J.A., Sanchez B., Rutkove S.B. (2017). Electrical Impedance Myography Detects Age-Related Muscle Change in Mice. PLoS ONE.

[61] Cheng K.-S., Su Y.-L., Kuo L.-C., Yang T.-H., Lee C.-L., Chen W., Liu S.-H. (2022). Muscle Mass Measurement Using Machine Learning Algorithms with Electrical Impedance Myography. Sensors.

[62] Akamatsu Y., Kusakabe T., Arai H., Yamamoto Y., Nakao K., Ikeue K., Ishihara Y., Tagami T., Yasoda A., Ishii K. (2022). Phase Angle from Bioelectrical Impedance Analysis Is a Useful Indicator of Muscle Quality. J. Cachexia Sarcopenia Muscle.

[63] Hobson-Webb L.D., Zwelling P.J., Pifer A.N., Killelea C.M., Faherty M.S., Sell T.C., Pastva A.M. (2018). Point of Care Quantitative Assessment of Muscle Health in Older Individuals: An Investigation of Quantitative Muscle Ultrasound and Electrical Impedance Myography Techniques. Geriatrics.

[64] Hobson-Webb L.D., Zwelling P.J., Raja S.S., Pifer A.N., Kishnani P.S. (2021). Quantitative Muscle Ultrasound and Electrical Impedance Myography in Late Onset Pompe Disease: A Pilot Study of Reliability, Longitudinal Change and Correlation with Function. Mol. Genet. Metab. Rep..

[65] Sato H., Nakamura T., Kusuhara T., Kenichi K., Kuniyasu K., Kawashima T., Hanayama K. (2020). Effectiveness of Impedance Parameters for Muscle Quality Evaluation in Healthy Men. J. Physiol. Sci..

[66] Kusche R., Ryschka M. (2020). Multi-Frequency Impedance Myography: The PhaseX Effect. IEEE Sens. J..

[67] Andringa A., van Wegen E., van de Port I., Kwakkel G., Meskers C. (2019). Measurement Properties of the NeuroFlexor Device for Quantifying Neural and Non-Neural Components of Wrist Hyper-Resistance in Chronic Stroke. Front. Neurol..

[68] Gong Z., Lo W.L.A., Wang R., Li L. (2023). Electrical Impedance Myography Combined with Quantitative Assessment Techniques in Paretic Muscle of Stroke Survivors: Insights and Challenges. Front. Aging Neurosci..

[69] Wang L.H., Sonbas Cobb B., Riem L., DuCharme O., Shaw D.W., Walker M., Eichinger K., Lewis L., Tawil R., Hamel J.I. (2025). Electrical Impedance Myography Captures Features of Muscle Structure Measured by MRI and Transcriptomic Analysis in Facioscapulohumeral Muscular Dystrophy. J. Neuromuscul. Dis..

[70] Kapravchuk V., Ishkildin A., Briko A., Borde A., Kodenko M., Nasibullina A., Shchukin S. (2025). Method of Forearm Muscles 3D Modeling Using Robotic Ultrasound Scanning. Sensors.

[71] Warneke K., Keiner M., Lohmann L.H., Brinkmann A., Hein A., Schiemann S., Wirth K. (2022). Critical Evaluation of Commonly Used Methods to Determine the Concordance between Sonography and Magnetic Resonance Imaging: A Comparative Study. Front. Imaging.

[72] Nescolarde L., Terricabras J., Mechó S., Rodas G., Yanguas J. (2020). Differentiation between Tendinous, Myotendinous and Myofascial Injuries by L-BIA in Professional Football Players. Front. Physiol..

[73] Hiyoshi T., Zhao F., Baba R., Hirakawa T., Kuboki R., Suzuki K., Tomimatsu Y., O’Donnell P., Han S., Zach N. (2023). Electrical Impedance Myography Detects Dystrophin-Related Muscle Changes in Mdx Mice. Skelet. Muscle.

[74] Kohler L., Puertollano R., Raben N. (2018). Pompe Disease: From Basic Science to Therapy. Neurotherapeutics.

[75] Albano D., Gitto S., Vitale J., Bernareggi S., Aliprandi A., Sconfienza L.M., Messina C. (2022). Comparison between Magnetic Resonance Imaging and Electrical Impedance Myography for Evaluating Lumbar Skeletal Muscle Composition. BMC Musculoskelet. Disord..

[76] Hamel J., Lee P., Glenn M.D., Burka T., Choi I.-Y., Friedman S.D., Shaw D.W.W., McCalley A., Herbelin L., Dimachkie M.M. (2020). Magnetic Resonance Imaging Correlates with Electrical Impedance Myography in Facioscapulohumeral Muscular Dystrophy. Muscle Nerve.

[77] Mesin L. (2020). Crosstalk in Surface Electromyogram: Literature Review and Some Insights. Phys. Eng. Sci. Med..

[78] Xu P., Zhou J., Zhuang Y., Li X., Vasic Z.L., Cifrek M., Liu Y., Gao Y. (2025). Static Force Prediction: Comparative Analysis and Fusion Strategies Between Surface Electromyography and Electrical Impedance Myography. IEEE Trans. Neural Syst. Rehabil. Eng..

[79] Zheng Z., Wu Z., Zhao R., Ni Y., Jing X., Gao S. (2022). A Review of EMG-, FMG-, and EIT-Based Biosensors and Relevant Human–Machine Interactivities and Biomedical Applications. Biosensors.

[80] Briko A., Kapravchuk V., Kobelev A., Hammoud A., Leonhardt S., Ngo C., Gulyaev Y., Shchukin S. (2021). A Way of Bionic Control Based on EI, EMG, and FMG Signals. Sensors.

[81] Cho Y., Kim P., Kim K.-S. (2020). Electrical Impedance Myography (EIM) for Multi-Class Prosthetic Robot Hand Control. Proceedings of the 2020 20th International Conference on Control, Automation and Systems (ICCAS), Busan, Republic of Korea, 13–16 October 2020.

[82] Nissler C., Mouriki N., Castellini C. (2016). Optical Myography: Detecting Finger Movements by Looking at the Forearm. Front. Neurorobot..

[83] Khant M., Gouwanda D., Gopalai A.A., Lim K.H., Foong C.C. (2023). Estimation of Lower Extremity Muscle Activity in Gait Using the Wearable Inertial Measurement Units and Neural Network. Sensors.

[84] Liu X., Zheng E., Wang Q. (2022). Real-Time Wrist Motion Decoding with High Framerate Electrical Impedance Tomography (EIT). IEEE Trans. Neural Syst. Rehabil. Eng..

[85] Iftime A., Scheau C., Babeș R.-M., Ionescu D., Periferakis A., Călinescu O. (2025). Confounding Factors and Their Mitigation in Measurements of Bioelectrical Impedance at the Skin Interface. Bioengineering.

[86] Rutkove S.B., Narayanaswami P., Berisha V., Liss J., Hahn S., Shelton K., Qi K., Pandeya S., Shefner J.M. (2020). Improved ALS Clinical Trials through Frequent At-home Self-assessment: A Proof of Concept Study. Ann. Clin. Transl. Neurol..

[87] Sonbas-Cobb B., Rutkove S.B., Wei B., Robicheau S., Arnold W.D., Hives K., Bartlett A., Riley P., Pagan A., Chrzanowski S.M. (2026). Multifrequency Electrical Impedance Myography Enhanced with Machine Learning for Screening Patients with Neuromuscular Disorders. Ann. Biomed. Eng..

[88] Pandeya S.R., Nagy J.A., Riveros D., Semple C., Taylor R.S., Hu A., Sanchez B., Rutkove S.B. (2022). Using Machine Learning Algorithms to Enhance the Diagnostic Performance of Electrical Impedance Myography. Muscle Nerve.

