# Supplementary material for: Surface Electrical Impedance Myography in Assessment of Morphofunctional Changes in Biological Tissues and Biofeedback Interfaces
Source: Sensors (Basel). 2026 Aug 4;26(15):4926. doi: 10.3390/s26154926 (PMC13468933; doi:10.3390/s26154926)
Supplement: Supplementary file 1 [file sensors-26-04926-s001.zip › sensors-4414324-supplementary.pdf]

PRISMA-ScR Checklist

Preferred Reporting Items for Systematic Reviews and Meta-Analyses Extension for Scoping Reviews (PRISMA-ScR)

Manuscript ID: sensors-4414324

Title: Surface Electrical Impedance Myography in the Assessment of Morphofunctional Changes in Biological Tissues and Biofeedback Interfaces: A Scoping Review

Authors: Vladislava Kapravchuk, Andrey Briko, Sergey Shchukin

Journal: Sensors (MDPI)

Checklist source: Tricco AC, Lillie E, Zarin W, et al. PRISMA Extension for Scoping Reviews (PRISMA-ScR): Checklist and Explanation. Ann Intern Med. 2018;169(7):467–473. doi:10.7326/M18-0850 — reference [45] of the manuscript.

Every row gives the section as well as the page, so each item can be found whatever the pagination of the typeset proof. Page numbers refer to the revised manuscript submitted with this checklist; where the two disagree, the section number is the one to trust.

SECTION	ITEM	PRISMA-ScR CHECKLIST ITEM	WHERE IT IS REPORTED
**TITLE**	1	**Title** *Identify the report as a scoping review.*	**p. 1.** The title ends with ": A Scoping Review", so the review type is clear before the reader opens the paper.
**ABSTRACT**	2	**Structured summary** *Provide a structured summary that includes (as applicable): background, objectives, eligibility criteria, sources of evidence, charting methods, results, and conclusions that relate to the review questions and objectives.*	**p. 1.** The abstract runs in the order this item asks for. It opens with what EIM is and why the signal is hard to interpret, states the aim, gives the publication window, languages and study designs we accepted, names Scopus as the source we searched, reports the 458 records screened and the 51 studies kept, and ends with the main conclusion and the gap it points to.
**INTRODUCTION**	3	**Rationale** *Describe the rationale for the review in the context of what is already known. Explain why the review questions/objectives lend themselves to a scoping review approach.*	**pp. 2–5, Section 1.** The Introduction lays out what is settled about EIM and where the evidence thins out. Why a scoping review in particular is argued in the last two paragraphs (pp. 4–5): the evidence on measurement parameters, individual anatomy, tissue condition and force-related deformation is scattered across single studies and has never been mapped as a whole. Mapping it is what a scoping review does; pooling effect estimates would not be meaningful here.
	4	**Objectives** *Provide an explicit statement of the questions and objectives being addressed with reference to their key elements (e.g., population or participants, concepts, and context) or other relevant key elements used to conceptualize the review questions and/or objectives.*	**p. 5, end of Section 1.** We state three questions outright: (1) which methodological factors affect how the EIM signal is interpreted; (2) how EIM relates to muscle morphology and function; (3) what limits the method today and where it should go next.<br><br>The PCC elements are given across Sections 1 and 2.1. **Population:** humans — healthy volunteers and patients — and animals. **Concept:** EIM-based assessment of morphofunctional change in muscle and the tissue around it. **Context:** diagnostics, rehabilitation, and biofeedback / bionic control interfaces.
**METHODS**	5	**Protocol and registration** *Indicate whether a review protocol exists; state if and where it can be accessed (e.g., a Web address); and if available, provide registration information, including the registration number.*	**p. 6, Section 2 (full statement); p. 5, end of Section 1 (short version).** We agreed a written protocol before running the search. It fixed the review questions, the PCC framework, the eligibility criteria, the Scopus string and the charting form, and Sections 2.1–2.4 reproduce it in full — the review can be repeated from the paper alone. A one-sentence version sits at the end of the Introduction so the reader meets the protocol before the methods rather than after.<br><br>**The review is not registered, and we say so plainly.** PROSPERO does not accept scoping reviews, and we did not use another registry, so there is no registration number. We considered depositing the protocol retrospectively and decided against it: any deposit would carry a date later than the completed review, which is less honest than simply reporting that no registration exists.<br><br>Nothing in the protocol changed while the review was running. The one addition was a handful of 2026 publications used as background during revision; we kept them out of the formal Scopus count and label them as such in Section 2.3.
	6	**Eligibility criteria** *Specify characteristics of the sources of evidence used as eligibility criteria (e.g., years considered, language, and publication status), and provide a rationale.*	**pp. 6–7, Sections 2.1 and 2.2.** We accepted peer-reviewed research articles, review articles in English, published 2016–2025. The ten-year window covers the current generation of EIM instrumentation — multichannel and multifrequency systems with modern signal processing — so that work done on superseded hardware would not skew the map.

SECTION	ITEM	PRISMA-ScR CHECKLIST ITEM	WHERE IT IS REPORTED
			Studies also had to report quantitative data tying impedance parameters to tissue properties, and to use a standard protocol: 1 kHz – 2 MHz, bipolar or tetrapolar.<br><br>**Section 2.2** says what we left out and why: cell cultures; in vitro needle-electrode work; whole-body BIA on its own; anything with no quantitative result; non-English publications; repeat papers on one sample; and measurements taken through clothing, bandages, gels of unknown conductivity, or over broken skin.
	7	**Information sources*** *Describe all information sources in the search (e.g., databases with dates of coverage and contact with authors to identify additional sources), as well as the date the most recent search was executed.*	**p. 6, Section 2; pp. 7–8, Section 2.3.** We searched one database, Scopus. The search first ran in December 2025 and was re-run in March 2026, which is the date of the most recent search.<br><br>We contacted no authors, and no grey literature, trial registries or reference-list chasing fed into the numbers in the flow diagram.<br><br>One database is a real constraint and we treat it as one — the choice is justified in Section 1 (p. 5) and its consequences are discussed in Section 4.2 (pp. 18–20).
	8	**Search** *Present the full electronic search strategy for at least 1 database, including any limits used, such that it could be repeated.*	**p. 7, Section 2.3.** The Scopus query is printed in full, with all three TITLE-ABS-KEY blocks and every limit: PUBYEAR > 2015 AND PUBYEAR < 2026; SUBJAREA restricted to MEDI, NURS, HEAL, VETE and NEUR; DOCTYPE to "ar" and "re"; LANGUAGE to English. It can be pasted into Scopus and re-run as printed.
	9	**Selection of sources of evidence** *State the process for selecting sources of evidence (i.e., screening and eligibility) included in the scoping review.*	**p. 6, Section 2; p. 7, Section 2.3.** Two stages: titles and abstracts first, then full text against the same criteria.<br><br>One author screened. The other two independently reviewed and validated both the selection and the approach, and we settled disagreements by discussion until all three of us agreed. We did not screen every record in duplicate, and we say so rather than describing this as double screening.
	10	**Data charting process** *Describe the methods of charting data from the included sources of evidence (e.g., calibrated forms or forms that have been tested by the team before their use, and whether data charting was done independently or in duplicate) and any processes for obtaining and confirming data from investigators.*	**pp. 8–9, Section 2.4.** Records went into Zotero 7.0.32 and were checked for duplicates. Charting used a form we agreed before charting began.<br><br>One author charted; the other two checked the entries independently, and discrepancies were resolved by consensus. We asked nothing of the original investigators — only what the published records report was charted.
	11	**Data items** *List and define all variables for which data were sought and any assumptions and simplifications made.*	**pp. 8–9, Section 2.4.** We charted:<br><br>• bibliographic details (type, authors, year, journal); • study objective; • design and methodology; • field of application (diagnostics, rehabilitation, muscle-function assessment, biofeedback); • sample characteristics; • tissue type and muscles examined;<br><br>• EIM technique parameters — impedance parameters used, probing-current frequency or range, bipolar vs. tetrapolar, electrode material and placement, interelectrode distance, measurement protocol;<br><br>• the reference method used for comparison (dynamometry, EMG, ultrasound, MRI, CT, functional testing);<br><br>• quantitative results tying EIM parameters to morphology and function — correlation coefficients, regression indices, and sensitivity to contractile force, fatigue, subcutaneous fat thickness, muscle cross-sectional area, adipose infiltration, atrophy and hypertrophy;<br><br>• the authors' own conclusions and stated limitations; whether they accounted for skin and subcutaneous fat; and whether there was external validation against a reference method.<br><br>The last two are charted on purpose: together with the technique parameters, they decide whether two studies can be compared at all.
	12	**Critical appraisal of individual sources of evidence** *If done, provide a rationale for conducting a critical appraisal of included sources of evidence; describe the methods used and how*	**Not done — stated on p. 6, Section 2.** We did not carry out a formal critical appraisal or risk-of-bias assessment of the individual sources. That follows PRISMA-ScR guidance and the point of the review: we set out to map the extent, range and nature of the evidence, not to estimate an intervention effect.

SECTION	ITEM	PRISMA-ScR CHECKLIST ITEM	WHERE IT IS REPORTED
		this information was used in any data synthesis (if appropriate).	Since we appraised nothing, nothing was weighted, ranked or excluded on appraisal grounds in the synthesis. We repeat the absence of appraisal among the limitations in Section 4.2, so it reads as a decision rather than an oversight.
	13	**Synthesis of results** *Describe the methods of handling and summarizing the data that were charted.*	**p. 9, Section 2.4; p. 9, opening of Section 3.** We summarized the charted data descriptively and narratively, grouped two ways: by measurement technique (electrode configuration, material, geometry, frequency protocol) and by the type of morphofunctional change detected. Quantitative relationships between EIM and reference measures are tabulated by reference method in Tables 1–4.<br>We ran no meta-analysis and pooled nothing. Electrode systems, frequency ranges and protocols differ too much across these studies for a pooled estimate to mean anything, and we give that reasoning in the text instead of leaving the absence unexplained.
**RESULTS**	14	**Selection of sources of evidence** *Give numbers of sources of evidence screened, assessed for eligibility, and included in the review, with reasons for exclusions at each stage, ideally using a flow diagram.*	**p. 8, Section 2.3, last paragraph, and Figure 1 (p. 8).**<br>Scopus returned 458 records and we found no duplicates. All 458 were screened on title and abstract, and 369 were excluded. 89 went to full text, of which 38 were excluded — review articles without primary EIM data, methodological or background studies, or not relevant to our scope. 51 studies remained. The text and Figure 1 carry the same numbers.
	15	**Characteristics of sources of evidence** *For each source of evidence, present characteristics for which data were charted and provide the citations.*	**pp. 9–16, Sections 3.1–3.3, with Tables 1–4.** Measurement technique, electrode configuration, frequency range, tissue and muscle examined, population and reference method are given for each source, with its citation, in the text and in the tables.
	16	**Critical appraisal within sources of evidence** *If done, present data on critical appraisal of included sources of evidence (see item 12).*	**Not applicable.** No critical appraisal was done — see item 12 and p. 6, Section 2.
	17	**Results of individual sources of evidence** *For each included source of evidence, present the relevant data that were charted that relate to the review questions and objectives.*	**pp. 12–16, Tables 1–4, with the text of Sections 3.2 and 3.3.**<br>Table 1 (pp. 12–13) — EIM parameters against recorded force. Table 2 (pp. 13–14) — EIM parameters against muscle tissue condition and morphofunctional change. Table 3 (p. 15) — EIM against MRI. Table 4 (pp. 15–16) — EIM against ultrasound.
	18	**Synthesis of results** *Summarize and/or present the charting results as they relate to the review questions and objectives.*	**pp. 9–16, Sections 3.1–3.3.** Section 3.1 answers question (1) — electrode configuration, material and geometry, frequency protocol, and the contribution of the skin–fat layer. Section 3.2 answers question (2) — how EIM parameters track muscle morphology and function.<br>Section 3.2 opens by separating time-resolved (dynamic) measurement from single-point measurement, because results from the two are not directly comparable and running them together is a common source of confusion in this literature.
**DISCUSSION**	19	**Summary of evidence** *Summarize the main results (including an overview of concepts, themes, and types of evidence available), link to the review questions and objectives, and consider the relevance to key groups.*	**pp. 16–20, Section 4, including 4.1 (p. 17) and 4.2 (p. 18).** Section 4 opens by tying the findings back to the gaps named in the Introduction, and the last paragraph of 4.2 does the same.<br>We discuss relevance for three groups: clinicians (diagnosis, tracking progression and treatment response, at-home assessment); rehabilitation specialists; and people building prostheses, orthoses, exoskeletons and proportional biofeedback interfaces. Section 4.1 deals with how EIM sits alongside EMG, force myography, ultrasound and MRI rather than replacing any of them.
	20	**Limitations** *Discuss the limitations of the scoping review process.*	**pp. 18–20, Section 4.2.** We state the limitations of the review process itself:<br>• we searched one database, so records indexed only in PubMed, Web of Science or elsewhere may have been missed; • only English-language publications were eligible; • electrode configurations, frequency ranges and protocols vary so widely that direct comparison is hard and pooling is not defensible; • we did not appraise the included sources; • we did not analyse animal-model studies comprehensively, and flag that as work for a separate review.<br>The single-database limitation also appears earlier, in Section 1 (p. 5), so a reader meets it before the results rather than after.

SECTION	ITEM	PRISMA-ScR CHECKLIST ITEM	WHERE IT IS REPORTED
**CONCLUSIONS**	**21**	**Conclusions** *Provide a general interpretation of the results with respect to the review questions and objectives, as well as potential implications and/or next steps.*	**pp. 20–21, Section 5.** We read the findings against the three questions and name concrete next steps: standardizing measurement protocols; finite-element modelling of current propagation in multilayer media with realistic geometry; simultaneous EIM and reference imaging (MRI, ultrasound) to quantify what each tissue contributes; hybrid systems combining EIM with EMG and dynamometry; flexible wearable electrode arrays; and a deliberately cautious line on machine learning until the signal generation mechanisms are better understood.
**FUNDING**	**22**	**Funding** *Describe sources of funding for the included sources of evidence, as well as sources of funding for the scoping review. Describe the role of the funders of the scoping review.*	**p. 21, Funding statement.** The review was funded by the Russian Science Foundation, grant 25-75-00100. The funder had no part in designing the review, in the search, selection, charting, synthesis or interpretation, in writing the manuscript, or in the decision to publish.<br><br>We did not chart the funding of the individual included sources. It has no bearing on our questions, which are about measurement methodology and tissue physiology rather than intervention effects — noted in Section 2 (p. 6).
